# Predictors of a Good Outcome after Endovascular Stroke Treatment with Stent Retrievers

**DOI:** 10.1155/2015/403726

**Published:** 2015-06-07

**Authors:** Ozcan Ozdemir, Semih Giray, Zulfikar Arlier, Demet Funda Baş, Yusuf Inanc, Ertugrul Colak

**Affiliations:** ^1^Department of Neurology, Eskisehir Osmangazi University Medical Faculty, Neurocritical Care, Cerebrovascular Disease, 26040 Eskisehir, Turkey; ^2^Department of Neurology, Baskent University Medical Faculty, Ankara, Turkey; ^3^Department of Biostatistics, Eskisehir Osmangazi University, Turkey

## Abstract

*Background and Purpose*. Successful recanalization after endovascular stroke therapy (EVT) did not translate into a good clinical outcome in randomized trials. The goal of the study was to identify the predictors of a good outcome after mechanical thrombectomy with stent retrievers. *Methods*. A retrospective analysis of a prospectively collected database included consecutive patients treated with stent retrievers. We evaluated the influence of risk factors for stroke, baseline NIHSS score, Alberta Stroke Program Early CT (ASPECT) score, recanalization rate, onset-to-recanalization and onset-to-groin puncture time, and glucose levels at admission on good outcomes. The number of stent passes during procedure and symptomatic hemorrhage rate were also recorded. A modified Rankin Scale (mRS) score of 0–2 at 90 days was considered as a good outcome. *Results*. From January 2011 to 2014, 70 consecutive patients with an acute ischemic stroke underwent EVT with stent retrievers. The absence of a medical history of diabetes was associated with good outcomes. Apart from diabetes, the baseline demographic and clinical characteristics of patients were similar between subjects with poor outcome versus those with good outcomes. Median time from onset to recanalization was significantly shorter in patients with good outcomes 245 (IQR: 216–313 min) compared with poor outcome patients (315 (IQR: 240–360 min); *P* = 0.023). Symptomatic intracranial hemorrhage was observed in eight (21.6%) of 37 patients with poor outcomes and no symptomatic hemorrhage was seen in patients with good outcomes (*P* = 0.006). In multivariate stepwise logistic regression analysis, a favorable ASPECT score (ASPECT > 7) and successful recanalization after EVT were predictors of good outcomes. Every 10-year increase was associated with a 3.60-fold decrease in the probability of a good outcome at 3 months. The probability of a good outcome decreases by 1.43-fold for each 20 mg/dL increase in the blood glucose at admission. *Conclusion*. To achieve a good outcome after EVT with stent retrievers, quick and complete recanalization and better strategies for patient selection are warranted. We need randomized trials to identify the significance of tight blood glucose control in clinical outcome during or after EVT.

## 1. Introduction

Early recanalization of occluded intracranial vessels is strongly associated with improved functional outcomes in patients with acute stroke treated with intravenous thrombolysis [1]. The overall recanalization rate was 46.2% with intravenous (IV) thrombolysis in a meta-analysis of 53 studies [2]. However, the recanalization rate is low in large vessel occlusions with IV thrombolysis. The complete recanalization rate is 10% in patients treated with IV thrombolysis for terminal internal carotid artery (ICA) occlusion and 31% in tandem ICA and middle cerebral artery (MCA) occlusion [3, 4]. Endovascular treatment (EVT) aims to increase the recanalization rate in patients with large vessel occlusion. Although recently published randomized trials have demonstrated better recanalization rates with the endovascular stroke treatment (EVT) compared to intravenous (IV) thrombolysis, successful recanalization did not translate into a better outcome with EVT [5–7]. However, in IMS III, SYNTHESIS expansion, and MR RESCUE trials, only a small proportion of patients were treated with new stent retrievers and this was criticized in these trials [5–7]. EVT with stent retrievers has achieved better recanalization and clinical outcomes when compared with MERCİ device and intra-arterial thrombolysis [8, 9]. Nevertheless, even in the stent retriever studies, a good outcome was achieved, no more than 60% of the patients [8, 9]. Therefore, patient selection is mandatory to have a good clinical outcome and for the avoidance of futile recanalization no matter what endovascular approach is used. The aim of our study is to identify the predictors of good outcome after EVT with new stent retrievers. Identifying predictors of good outcome may help us to improve the outcome after acute stroke endovascular treatment.

## 2. Materials and Methods

We performed a retrospective study of consecutive patients with acute ischemic stroke who underwent EVT with new stent retrievers between January 1, 2011, and February 1, 2014, at Eskisehir Osmangazi University Stroke Center and at Adana Baskent University, Department of Neurology. From 2011 to 2014, 2500 acute stroke patients were admitted to two stroke centers. Two hundred of 2500 (8%) acute stroke patients were eligible for recanalization treatment including intravenous thrombolysis and endovascular treatment. Intravenous fibrinolysis was initiated in 110 of 200 patients. EVT was performed in 90 patients. Among 90 patients, 10 patients received intra-arterial thrombolysis alone and the penumbra mechanical thrombectomy system was performed in 10 patients. Seventy acute ischemic stroke patients who underwent new stent retrievers were included in the analysis. The local ethics committee approved the analysis and data collection.

## 3. Patient Selection

All patients were evaluated by a stroke neurologist and underwent cranial CT scanning without contrast. Endovascular treatment with new stent retrievers was initiated within 6 h of symptom onset for the anterior circulation stroke and 8 h of symptom onset for the basilar thrombosis. Patients aged 18–80 years presenting with moderate-to-large strokes (NIHSS ≥ 10) in the setting of an angiographically (digital subtraction angiography) proven occlusion of a proximal intracranial artery (e.g., internal carotid artery, middle cerebral artery M1 and/or M2 segments, and basilar or vertebral arteries) were potential candidates for EVT. In accordance with institutional stroke protocol, moderate-to-severe acute stroke patients presenting with 4.5 h after onset received IV rtPA (0.9 mg/kg over 40 minutes) and were examined by a stroke neurologist. In the presence of dramatic clinical improvement (NIHSS ≥ 8), a full dose of IV fibrinolysis was administered and EVT was not considered. If no dramatic clinical improvement was observed after IV rt-PA, patients transferred to neuroangiography suite for EVT. With this protocol, we detected major intracranial vessel occlusion in all of our patients on DSA (Figure 2). In patients with anterior circulation stroke, the presence of early infarct signs within the MCA territory was assessed on baseline CT scans using the Alberta Stroke Program Early Computed Tomography (ASPECT) score [10]. Patients with an evidence of intracranial hemorrhage or major ischemic infarction (acute ischemic change in more than a third of the middle cerebral artery territory or having an Alberta Stroke Program Early CT score of <5) were excluded from EVT. For posterior circulation strokes, patients with extensive brainstem lesions (e.g., bilateral pons or mesencephalic involvement) were excluded from EVT. Patients who had contraindication to IV fibrinolysis or presenting between 4.5 and 6 h for anterior circulation strokes or 4.5 and 8 h for posterior circulation strokes were treated with stand-alone thrombectomy. Patients with known coagulopathy or systemic bleeding disorder and a prestroke score on the mRS ≥ 2, which could affect the outcome, were excluded from the analysis. Details of the inclusion and exclusion criteria for EVT were given in Table 1.

## 4. Outcome Measures and Clinical Assessment

Clinical severity at baseline and 24 h after symptom onset was assessed prospectively by using the National Institutes of Health Stroke Scale (NIHSS) conducted by the stroke neurologist. A good outcome was defined as a score of 0 to 2 on the modified Rankin Scale (mRS) at 90 days. A poor outcome was defined as a score of 3 to 6 on the mRS at 90 days. Subsequent NIHSS recordings were collected at 1 and 24 hours after the EVT. Dramatic recovery was defined as an NIHSS score of 0 to 3 at 24 hours or a decrease of ≥10 points in the NIHSS score at 24 hours [11]. Stroke type was determined using the Trial of Org 10172 in Acute Stroke Treatment (TOAST) trial criteria after a diagnostic work-up was completed [12]. The extent of hypodensity on baseline noncontrast CT (NCCT) was quantified as described in ASPECT score. ASPECT score was presented as dichotomized into ≤7 and >7 [13]. We determined the cutoff point and dichotomization for ASPECT score based on clinical judgement and previous literature [13]. All patients had a CT or MRI scan 24 hours after the EVT.

## 5. Interventional Treatment

All procedures were performed on a monoplane flat detector angiography machine (Siemens Axiom Artis, Siemens Healthcare, Erlangen, Germany) under conscious sedation. Stent retrievers were used as first-line device. The Revive device (Codman endovascular) was used in 15 cases, the Solitaire FR device (Covidien, Irvine, California) was used in 14 cases, Trevo (Stryker, Kalamazoo, Michigan) was used in 10 cases, and pREset (Phenox GmbH, Bochum, Germany) was used in 31 cases. In the anterior circulation, a 6F guiding catheter or a long 6F sheath (Neuron MAX, Penumbra, Inc., Alameda) was placed in the internal carotid or common carotid artery. In some cases, a triple coaxial system consisting of a long sheath, a five-French intermediate catheter, and a 0.021-inch microcatheter was used. For the posterior circulation, a 6F Envoy guiding catheter (Codman endovascular) was placed through a sheath into the vertebral artery. Cervical access vessel occlusion or stenosis was initially treated with angioplasty. In case of persistent cervical vessel occlusion despite angioplasty, stenting was performed after giving 600 mg clopidogrel and 600 mg acetylsalicylic acid via a nasogastric tube. Except patients who underwent emergency carotid stenting, all patients received 100 mg ASA before EVT in the emergency department. During interventional stroke procedure, 2000 units of bolus heparin were given routinely. A 0.021-inch inner lumen microcatheter was navigated distal to the point of occlusion over a 0.014-inch guidewire. The stent retriever advanced through the microcatheter. After deployment, the stent retriever was maintained in place for approximately 5 minutes. The microcatheter and the fully deployed stent retriever pulled back together under continuous manual aspiration with 50 mL syringe into the guiding catheter or distal access catheter. The modified thrombolysis in cerebral infarction (TICI) score was used to evaluate recanalization results. Successful recanalization was defined as TICI 2b or 3. The treatment was considered a failure if the target vessel was not successfully recanalized with a maximum of 4 passes with stent retrievers. No antiplatelet or heparin was administered within 24 h of procedure. A CT or MRI was performed 24 hours after the procedure. If no hemorrhage was present, aspirin 300 mg/day was given. In the study, the time from symptom onset to groin puncture (OTP) and from onset to recanalization (ORT) and the number of stent deployments were recorded.

## 6. Complication Definitions

Hemorrhagic transformations were classified according to radiological and clinical criteria [13, 14]. Hemorrhagic infarction 1 (HI1) was defined as small petechiae along the margins of the infarct and HI2 was defined as confluent petechiae within the infarcted area but no space occupying effect. Parenchymal hematoma 1 (PH1) was defined as blood clots in ≤30% of the infarcted area with some slight space-occupying effect, and PH2 was defined as blood clots in >30% of the infarcted area with a substantial space occupying effect. Symptomatic intracerebral hemorrhage was defined as local or remote PH2 or PH1 on the 24-hour postprocedural CT scan combined with an increase of ≥4 NIHSS points from baseline or leading death [13, 14]. We reported the rate of subarachnoid or intraventricular hemorrhage.

## 7. Statistical Analysis

The Kolmogorov-Smirnov test for normality and the equal variance test were performed before any statistical analysis was used. Bivariate comparisons were made using *χ* ^2^ exact tests for categorical, Student's *t*-test was used for continuous variables, and the Mann-Whitney *U*-test was used for ordinal variables and continuous variables that were not normally distributed. Univariate analysis was performed to compare the outcome, baseline characteristics, procedural parameters, and complications of patients with good outcome and patients with poor outcome after EVT. For multivariate analysis, logistic regression was used to assess the effect of clinical, neuroimaging, procedural parameters on good outcomes (mRS 0–2) with a backward inclusion model. The goodness-of-fit of the models was assessed using Hosmer and Lemeshow *χ* ^2^ test. The significance level was set at *P* < 0.05. Statistical analysis was performed using SPSS version 19 (IBM SPSS Statistics; SPSS Inc., Chicago, IL, USA).

## 8. Results

### 8.1. Patient Characteristics

During the study period, 70 consecutive (29 female and 41 male; mean age 57 ± 10.4) patients with acute occlusion of intracranial large vessels occlusions underwent EVT with stent retrievers. Detailed patient baseline characteristics, procedural parameters, and target vessels are summarized in Table 1.

### 8.2. Procedural Results

Thirty-three of 70 patients (47%) received IV thrombolysis before EVT. Fifteen patients (21.4%) received intra-arterial rt-PA and mechanical thrombectomy. After IV thrombolysis, combined intra-arterial rt-PA and mechanical thrombectomy was performed in 13 of 70 patients (18.6%). Stent retriever alone was performed in 21 of 70 patients (30%). Fifty-nine of 70 patients (84%) had anterior circulation stroke (M1, 34 (48.6%); M2, 5 (7.1%); Carotid T, 10 (14.3%); MCA/ICA tandem occlusion 10 (14.3%)). Eleven out of 20 patients with concomitant cervical carotid occlusion were treated with only angioplasty and 8 patients underwent both stenting and angioplasty prior to an intracranial recanalization procedure. Manual aspiration was performed in one patient for the cervical carotid occlusion followed by stent retriever deployment for MCA occlusion. One patient with proximal MCA occlusion and one patient with carotid T occlusion were treated with angioplasty due to persistent intracranial stenosis after the deployment of stent retrievers. Apart from these patients who underwent angioplasty and stenting, no patients received adjuvant thrombectomy device including the penumbra aspiration system, angioplasty, or permanent stenting. Eleven patients (16%) had basilar thrombosis leading to posterior circulation stroke. Successful recanalization (TICI scores of 2b and TICI 3) was achieved in 47 (67%) of 70 patients. Successful recanalization rates did not differ significantly between anterior (69%) and posterior (64%) circulation vessel occlusions (*P* > 0.05). Recanalization rates were 76% (25 of 33) and 60% (22 of 37) in patients with or without concomitant IV thrombolysis, respectively (*P* = 0.232). Symptomatic hemorrhage was observed in 8 patients (11.8%). Posttreatment imaging revealed 6 (8.6%) PH1 cases and 4 (5.7%) PH2 cases. Two patients (2.85%) had both diffuse SAH and PH2. Five patients (7.1%) had asymptomatic focal SAH. Symptomatic hemorrhage rates were 12.1.% (4 of 33) and 10.8 (4 of 37) in patients with or without concomitant IV thrombolysis, respectively (*P* < 0.05). Administration of intravenous or intra-arterial rt-PA in patients who underwent EVT did not affect the symptomatic hemorrhage rate. Symptomatic hemorrhage was observed in 2 patients in the stand-alone thrombectomy group and 1 in patients who received IV thrombolysis and EVT, 3 in patients who received IV thrombolysis, intra-arterial rt-PA, and EVT, and 2 in patients who received intra-arterial rt-PA and EVT (*P* = 0.425).

### 8.3. Predictors of Good Outcome

Overall, thirty-seven patients (53%) had poor outcomes (mRS 3–6) and 33 patients (47%) had good outcomes (mRS 0–2) at 3 months. Tables 2 and 3 give the detailed results on the univariate and multivariate analysis of potential factors predicting good clinical outcomes at three months. Univariate analysis was done to compare the baseline characteristics and procedural parameters of patients with good outcomes and poor outcomes at three months. No differences were found in sex, medical history of smoking, hypertension, dyslipidemia, atrial fibrillation, and baseline NIHSS score between patients with good outcomes and those with poor outcomes (Table 2). The absence of a medical history of diabetes was associated with good outcome (*P* = 0.022). The mean age was significantly lower in patients with good outcomes compared with poor outcome patients (60 ± 8.8 versus 54 ± 11.2; *P* = 0.012). Patients with good outcomes had significantly lower baseline glucose levels than those with poor outcomes (127 ± 38.5 versus 187 ± 11.2; *P* < 0.001). Among patients with anterior circulation stroke, twenty-six of the 42 patients (62%) with ASPECT > 7 and 4 of 13 patients (23.5%) with ASPECT ≤ 7 had a good outcome after EVT (*P* = 0.017). Administration of IV thrombolysis prior to EVT did not have influence on the outcome in the analysis (*P* = 0.158). Twenty-five of 29 patients (86.2%) who had a dramatic recovery at 24 hours achieved good clinical long-term outcome and only eight of 41 patients (19.5%) who did not have dramatic recovery achieved good clinical long-term outcome (*P* < 0.001). Twenty-eight of 33 patients (85%) patients with good outcome achieved successful recanalization as compared to 19 of 37 patients (51.4%) with poor outcome (*P* = 0.006). The median OTP time was non-statistically significantly shorter in patients with good outcomes 187 (IQR: 150–240) compared with those with poor outcomes (240 (IQR: 180–300); *P* = 0.088). Median time from onset to the achievement of recanalization was significantly shorter in patients with good outcomes 245 (IQR: 216–313) compared with poor outcome patients (315 (IQR: 240–360); *P* = 0.023). Twenty-five of 32 patients (78%) had good clinical outcome if symptom onset-to-recanalization time was ≤5 hours. However, in the presence of symptom-to-recanalization time beyond 5 hours, only nine of 23 patients (39%) had a good outcome despite successful recanalization after EVT. The median number of passes with stent retrievers was significantly lower, 1 (IQR: 1-2), in patients with good outcome than in those with poor outcome (2 (IQR: 2-3); *P* = 0.008). Only 11 of 24 patients (46%) achieved complete recanalization if more than two passes with stent retrievers were required compared with patients who required ≤2 attempts (78%; *P* = 0.013). Nine of 11 basilar thrombosis patients (81%) and 15 of 59 patients (25%) with anterior circulation vessel occlusions required more than >2 passes with stent retrievers (*P* = 0.001). Symptomatic intracranial hemorrhage occurred in eight (21.6%) of 37 patients with poor outcome and no symptomatic hemorrhage was observed in patients with good outcome (*P* = 0.006). Patients with symptomatic intracranial hemorrhage had higher admission glucose levels compared with those without hemorrhage (202 ± 85 versus 153 ± 58; *P* = 0.038).

In multivariable logistic regression analysis, including the admission NIHSS score, OTP time, age, serum glucose levels, and recanalization, dichotomized ASPECT was performed. As shown in Table 3, age, serum glucose levels, presence of successful recanalization, and ASPECT > 7 on CT were predictors of good clinical outcomes. Every 10-year age increase was associated with a 3.60-fold decrease in the probability of a good functional outcome at 3 months. The probability of a good outcome decreases by 1.43-fold for each 20 mg/dL increase in the admission blood glucose. Recanalization was the strongest independent predictor of a good outcome (OR: 48.6; 95% CI: 4.2–559; *P* = 0.002).

## 9. Discussion

This study including analysis from two centers assessed the predictors of good clinical outcomes of patients being treated with stent retrievers. We have identified that a favorable baseline CT (ASPECT score > 7), and successful recanalization (TICI 2b-3) are independent predictors of good clinical outcomes (Table 4). Older age and higher glucose levels have a negative impact on clinical outcome. In addition, shorter symptom-onset-to-recanalization time and reduced numbers of stent deployment during the procedure are associated with good clinical outcome.

There is compelling evidence that a good clinical outcome is strongly correlated with successful recanalization [2]. In the present study, we found that complete recanalization is the most powerful independent predictor of good clinical outcomes after EVT with stent retrievers. The IMS III study showed no benefit of endovascular procedures over standard IV thrombolysis; however, the rate of successful recanalization (TICI 2b-3) was <45% [6]. The lower rate of successful recanalization in IMS III may be related to less use of newer technologies such as stent retriever. The Solitaire With the Intention for Thrombectomy (SWİFT) trial and the TREVO II trial compared new technology stent retrievers with a first-generation MERCİ retriever and have shown the superiority of stent retrievers over MERCİ retriever in achieving successful revascularization [8, 15]. Since TICI and modified TICI are superior to TIMI for evaluating tissue reperfusion and predicting clinical outcome, TICI scale was used in our study [16, 17]. Unlike the SWIFT trial, which has used the TIMI grading scale, recanalization was assessed with the TICI scale in the TREVO II trial [8]. In the TREVO II trial, 68% of the patients achieved successful recanalization (core laboratory, TICI ≥ 2b). In line with TREVO II trial, successful recanalization rate was 67% in our study [15].

In present the study, time to recanalization was associated with good outcomes. Moreover, good clinical outcomes were observed in 78% of patients after EVT if symptom-onset-to-recanalization time was ≤5 hours. A recent analysis of pooled data from the MERCİ, TREVO, and TREVO II trials showed an 11% increase in the odds of functional dependence in every 30-minute delay from stroke onset to endovascular treatment [18]. Furthermore, previous studies have shown that onset-to-reperfusion time has a great impact on mortality, functional outcomes, and intracerebral hemorrhage rates [18–21]. A prospective multicenter registry of patients with basilar artery occlusion showed an association between early recanalization and a more favorable outcome and all patients with severe stroke treated >9 hours after symptom onset did not benefit from recanalization therapy [22]. In the endovascular arm of the IMS III trial, important delays were demonstrated prior to reperfusion.

Several studies have shown that baseline core infarct size is an important predictor of endovascular treatment outcomes [23, 24]. MR diffusion-weighted imaging is the most accurate method to identify infarct core; however, NCCT remains the most commonly used neuroimaging modality [24]. Using ASPECT score to rate ischemic change on NCCT may provide a systematic method to quantify early ischemic changes in the brain due to acute ischemic stroke in the anterior circulation [25, 26]. In the present study, a favorable baseline CT scan (ASPECT > 7) was independent predictor of a 90-day good clinical outcome after EVT with stent retrievers. Moreover, 62% of anterior circulation stroke patients with baseline favorable CT scans achieved good clinical outcomes. In a large study of patients with anterior circulation stroke treated with the Penumbra system supports the importance of pretreatment NCCT ASPECT score in predicting clinical outcomes after EVT. Higher ASPECT score was associated with reduced mortality and better functional outcomes [10]. Moreover, patients with favorable baseline CT scans (ASPECT > 7) were almost twice as likely to achieve a favorable outcome in the IMS III trial [27].

In this study, younger age was an independent predictor of good clinical outcomes. In addition, every 10-year age increase was associated with a 3.6-fold decrease in the probability of good outcome. In a multicenter study of endovascular treatment of anterior circulation stroke, outcome was highly dependent on patients' age. Good clinical outcomes were observed in 60% of the patients within the lowest age quartile (range: 18–56 years). In contrast, only 37% of patients within the range between 69 and 76 years had good clinical outcomes. Furthermore, even in the absence of any hemorrhagic complications and after the exclusion of patients with prestroke disability, older age exists as a predictor of poor outcomes despite successful recanalization [28]. A recent analysis of pooled data confirms the dramatic impact of age on outcomes after EVT. Every 10-year age increase was associated with a 92% relative increase in the odds of functional dependence after EVT with a TREVO thrombectomy device. The proportion of functional dependence was 82% in those aged >80 years and only 28% in people who aged ≤60 years [18]. Reduced plasticity, impaired collateral circulation, higher frequency of prestroke comorbid conditions and poststroke medical complications, and difficult vessel anatomy may contribute to reduced functional outcomes in older age [28]. Although advanced age has some negative effect on EVT outcome, it is important to consider all predictors of good clinical outcomes globally, rather than excluding patients from EVT only because of advanced age.

Our analysis demonstrated a 1.43-fold decrease in the probability of a good outcome for each 20 mg/dL increase in blood glucose at admission. Furthermore, admission glucose level was significantly associated with increased risk of symptomatic ICH after EVT with stent retrievers. In a registry and systematic review, admission blood glucose and history of diabetes mellitus were associated with poor clinical outcomes and increased risk of ICH in patients treated by IV and/or intra-arterial therapy [29]. Moreover, a multicenter trial evaluated the prognostic significance of blood glucose at admission and change in blood glucose at 48 hours from the baseline value in diabetic and nondiabetic patients before and after EVT. The failure of blood glucose decrease in the first 48 hours (glucose level drop > 30 mg/dL) and higher blood glucose levels (glucose ≥ 116 mg/dL) were both significant predictors of poor outcome and death. Only higher glucose levels at admission were associated with poor outcomes in diabetic patients [30]. There are several explanations regarding the contribution of higher glucose levels to poor outcomes and ICH. Hyperglycemia is associated with larger infarct volumes and reduced salvage of perfusion-diffusion mismatch tissue [31]. On the other hand, hyperglycemia may cause a larger increase of the infarct volume leading to a worse clinical outcome despite recanalization [32]. Increased risk of ICH in patients with hyperglycemia after EVT may be due to the blood-brain barrier disruption and microvasculature impairments [33, 34].

In the study, the median number of stent deployment was lower in good outcome patients. On the other hand, patients who required more passes (number of stent deployment > 2) with stent retrievers were less likely to achieve successful recanalization. A larger clot extent and proximal clot location causing resistant clots may explain the association between lower recanalization rates and the need for more passes with stent retrievers. The majority of patients with basilar thrombosis required more than 2 passes with stent retrievers during EVT. Thus, additional endovascular strategies such as the Penumbra aspiration system may be used in these patients.

**Figure 1:**
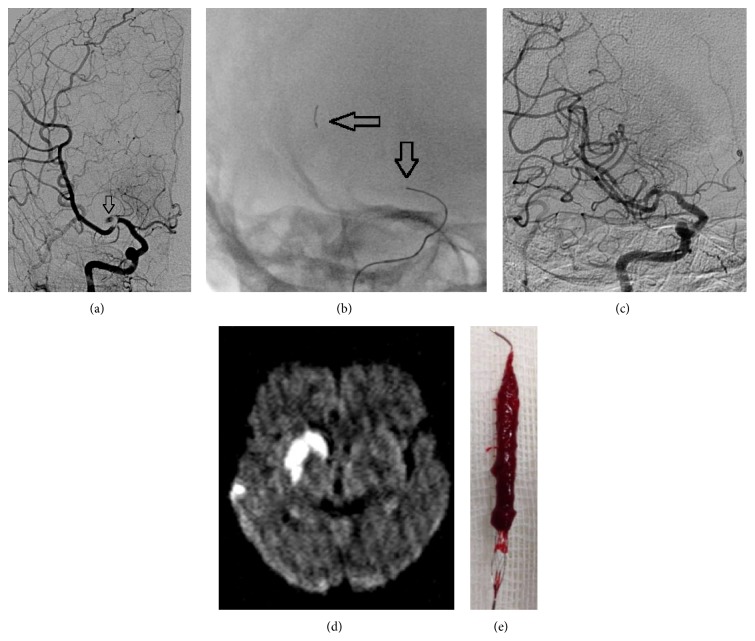
(a) Angiogram shows a thrombus on proximal MCA and an occlusion of superior division of MCA. (b) Deployed REVİVE device (distal and proximal markers = black arrow). (c) After retrieval of the stent, the vessel is recanalized to a TICI-3 state. (d) Control MRI scan shows right hemispheric striatocapsular infarction and multiple parietal small embolic infarctions. At 3 months, patients had a good outcome. (e) Large thrombus is adherent to the stent struts.

**Figure 2:**
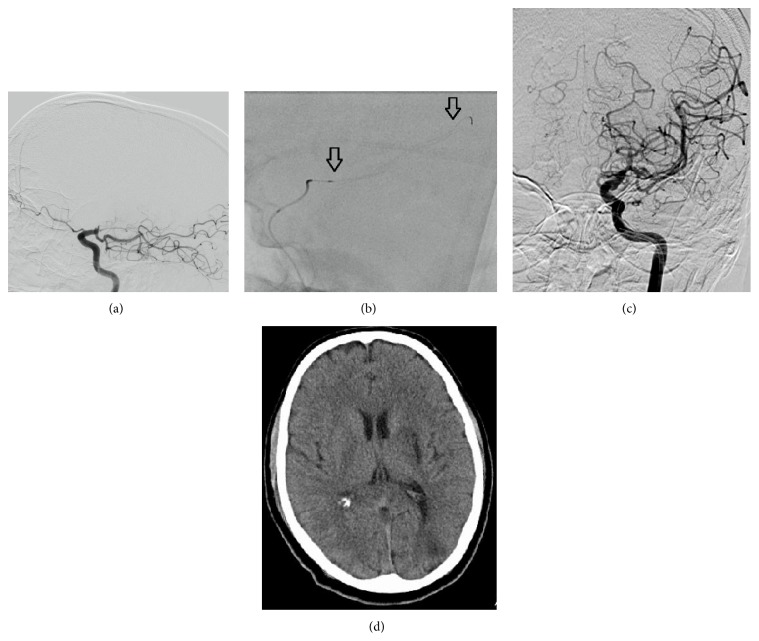
(a) DSA reveals persistent carotid T occlusion and fresh thrombus on the occluded segment. Fetal PCA is observed on the lateral projection (black arrow). (b) Deployed Trevo-ProVue device (distal and proximal markers = black arrow). (c) After two passes with Trevo device, successful recanalization was achieved (TICI 2b). Both anterior cerebral arteries are filled from right hemisphere. (d) Control CT scan shows small infarction on basal ganglia.

Our study has several limitations. The retrospective nature of the study is a limitation. Data were extracted from a prospectively collected database, but the angiograms were retrospectively analyzed (Figure 1). The overall number of patients analyzed is small and thus may reduce the significance of our statistical analysis. We did not consider EVT in patients with advanced age (≥80 years); therefore, our results may not be fully representative of the entire stroke population. Posterior circulation stroke patients were included in the analysis and the bias may be introduced since NIHSS score provides limited information. Furthermore, the associations of collateral flow and thrombus length with good outcomes have not been investigated systematically in this analysis.

## 10. Conclusion

There are multiple factors that determine the predictors of good clinical outcome in patients who underwent endovascular treatment with stent retrievers. It is important to achieve quick and higher rates of recanalization to improve good outcome. Hence, reducing the delays before or during endovascular stroke procedure is recommended. History of diabetes and higher admission glucose levels are inversely related to good outcome. Randomized trials are warranted to delineate the significance of tight blood glucose control on clinical outcome in the setting of endovascular stroke treatment. Clearly, in a time period, a large number of RCTs are underway; the only sensible course of action for a self-respecting intervention center is joining a multicenter trials. If not possible, the second best one would be to meticulously register all patients and adhere to protocols, until either trials have convincingly proven the absence of the presence of a clinically relevant treatment effect.

## Figures and Tables

**Table 1:** Patient selection criteria for endovascular stroke treatment with stent retrievers.

Inclusion and exclusion criteria	
*Clinical inclusion criteria* Age: 18–80 years Patients who presented within 6 h of symptom onset for the anterior circulation stroke Patients who presented within 8 h of symptom onset for the basilar thrombosis An NIHSS ≥10 at the time that I.V rt-PA is begun and if no dramatic clinical improvement was observed after IV rt-PA Patients who had contraindication to IV rt-PA and NIHSS ≥10 at the admission	

*Clinical exclusion criteria* Moderate to severe stroke patients who had dramatic clinical improvement (NIHSS ≥8) after IV rt-PA History of severe allergy to contrast medium or nitinol Patients with a preexisting neurological disease that cause moderate disability (mRS ≥2) Advanced and terminal illness Presumed septic embolus or suspicion of bacterial endocarditis Clinical presentation suggests a subarachnoid hemorrhage even if the initial CT scan is normal Baseline lab values: glucose <50 mg/dL or >400 mg/dL, platelets <100 000	

*Imaging exclusion criteria* CT evidence of intraparenchymal tumor CT evidence of intracranial hemorrhage Large (more than 1/3 of the middle cerebral artery) regions of clear hypodensity on baseline CT or having ASPECT score of <5 MRI or CT evidence of extensive brainstem lesions (e.g., bilateral pons or mesencephalic involvement)	

**Table 2:** Baseline characteristics and procedural parameters of all patients.

Characteristics	
Age, y	57.4 (10.4)
Female sex, *n* (%)	29 (41.4)
NIHSS score on admission, median (IQR)	20 (18–22)
Vascular risk factors, *n* (%)	
Hypertension	42 (60)
Diabetes mellitus	21 (30)
Atrial fibrillation	24 (34)
Dyslipidemia	41 (59)
Current smoking	32 (46)
Stroke etiology, *n* (%)	
Cardioembolism	34 (49)
Large artery disease	27 (38)
Other determined etiology	2 (3)
Unknown etiology	7 (10)
ASPECT >7, *n* (%)	42 (71)
Occlusion site, *n* (%)	
Carotid T occlusion	10 (14)
MCA/ICA tandem occlusion	10 (14)
M1 middle cerebral artery	34 (49)
M2 middle cerebral artery	5 (7)
Basilar thrombosis	11 (16)
Time issues	
Onset to groin puncture, min	205 (180–251)
Onset to recanalization, min	270 (240–340)
Successful recanalization, *n* (%)	
(TICI 2b, 3)	47 (67)
Outcome, *n* (%)	
Modified Rankin Scale: 0–2	33 (47)
Modified Rankin Scale: 3–6	37 (53)
Mortality	19 (27)
Dramatic recovery	29 (41)
Symptomatic ICH, *n* (%)	8 (11.4)

Values are mean (SD), median (IQR), or *n* (%) as appropriate.

NIHSS: National Institutes of Health Stroke Scale, mRS; Modified Rankin Score, ASPECT; Alberta Stroke Program Early CT Score for MCA territory stroke, and ICH; intracerebral hemorrhage.

A two-sided *P* value <0.05 was considered statistically significant. NS: not significant.

**Table 3:** Predictors of good clinical outcome.

Variable	Good outcome *n* = 33	Poor outcome *n* = 37	*P*
Age,y	53.8 ± 2.0	60.7 ± 2.0	0.012
Female sex, *n* (%)	13.8 (39.4)	16 (43.2)	0.934
NIHSS score on admission, median (IQR)	20 (18–22)	21 (20–22.5)	0.064
Vascular risk factors, *n* (%)			
Hypertension	17 (51.5)	25 (67.6)	0.261
Diabetes mellitus	5 (15.2)	16 (43.2)	0.022
Atrial fibrillation	10 (30.3)	14 (37.8)	0.681
Dyslipidemia	18 (54.5)	23 (62.2)	0.687
Current smoking	17 (51.5)	15 (40.5)	0.497
Cardioembolic stroke etiology, *n* (%)	13 (39.3)	20 (54)	0.324
Large artery disease, *n* (%)	14 (51.8)	19 (44.1)	0.704
ASPECT >7, *n* (%)	26 (87)	16 (55.2)	0.017
Admission glucose, mg/dL	126 ± 38.5	187 ± 67.6	0.002
Occlusion site, *n* (%)			
Carotid T occlusion	2 (21.6)	8 (6.1)	0.09
MCA/ICA tandem occlusion	5 (13.2)	5 (15.2)	1.00
Basilar thrombosis	3 (9.1)	8 (21.6)	0.267
Time issues			
Onset to groin puncture, min	187.5 (150–240)	240 (180–300)	0.088
Onset to recanalization, min	245 (216–314)	315 (240–360)	0.023
Onset to recanalization >5 h^*^	7 (22)	14 (61)	0.008
Successful recanalization, *n* (%)			
(TICI 2b, 3)	28 (85)	19 (51.4)	0.006
Number of stent deployment	1 (1-2)	2 (2-3)	0.008
IV thrombolysis, *n* (%)	19 (57.6)	14 (37.8)	0.158
Symptomatic ICH, *n* (%)	0 (0)	8 (21.6)	0.006

Values are mean (SD), median (IQR), or *n* (%) as appropriate.

NIHSS: National Institutes of Health Stroke Scale, mRS: modified Rankin Score, ASPECT; Alberta Stroke Program Early CT Score for MCA territory stroke, and ICH: intracerebral hemorrhage.

A two-sided *P* value <0.05 was considered statistically significant. NS: not significant.

^*^Patients who achieved successful recanalization were included in the analysis.

**Table 4:** Results of logistic regression to find independent predictors of good clinical outcome.

	OR	95% CI	*P*
Age, years	0.893	0.805–0.990	0.032
Glucose, mg/dL	0.98	0.962–0.996	0.017
ASPECT >7, *n*	9.63	1.34–69	0.024
Successful recanalization, *n*	43.8	3.314–580	0.004

NIHSS: National Institutes of Health Stroke Scale and ASPECT: Alberta Stroke Program Early CT Score for MCA territory stroke. Onset-to-groin puncture time and NIHSS score were included in the analysis and did not predict good outcome.
